# Docetaxel Resistance in Breast Cancer: Current Insights and Future Directions

**DOI:** 10.3390/ijms26157119

**Published:** 2025-07-23

**Authors:** Fátima Postigo-Corrales, Asunción Beltrán-Videla, Antonio David Lázaro-Sánchez, Ana María Hurtado, Pablo Conesa-Zamora, Ana Belén Arroyo, Ginés Luengo-Gil

**Affiliations:** 1 Health Sciences Faculty, Universidad Católica de Murcia (UCAM), 30107 Guadalupe, Spain; fpostigo@ucam.edu (F.P.-C.); amhurtado@ucam.edu (A.M.H.); barroyo@ucam.edu (A.B.A.); 2 Group of Molecular Pathology and Pharmacogenetics, Pathology and Clinical Analysis Department, Instituto Murciano de Investigación Biosanitaria (IMIB), Hospital General Universitario Santa Lucía, 30202 Cartagena, Spain; asuncionbeltranvi@gmail.com; 3 Department of Medical Oncology, Morales Meseguer General University Hospital, 30008 Murcia, Spain; anlazaro@hotmail.com

**Keywords:** breast cancer, docetaxel, taxanes, resistance to chemotherapy

## Abstract

Docetaxel is a chemotherapeutic agent widely used for breast cancer treatment; however, its efficacy is often limited by drug resistance and associated toxicity. This review examines the molecular mechanisms of docetaxel resistance in breast cancer and discusses research advances and future directions for overcoming this challenge. Key resistance mechanisms include alterations in drug targets (microtubules), increased drug efflux, suppression of apoptosis, activation of survival signalling pathways, epithelial-to-mesenchymal transition (EMT), and cancer stem cell enrichment. An evolutionary perspective distinguishes between intrinsic and acquired resistance, emphasising the need for adaptive therapeutic strategies. Recent advances in genomic profiling, non-coding RNA research, novel drug combinations, and biomarker-guided therapies have also been reviewed. Emerging approaches, such as targeting the tumour microenvironment, harnessing immunotherapy, and implementing adaptive dosing schedules, have been discussed. This review emphasises the understanding of resistance as a multifactorial phenomenon that requires multipronged interventions. Research has aimed to identify predictive biomarkers, develop targeted agents to reverse resistance, and design rational combination strategies to improve patient outcomes. Progress in deciphering and targeting docetaxel resistance mechanisms holds promise for enhancing treatment responses and extending survival in patients with breast cancer.

## 1. Introduction

**Figure 1:**
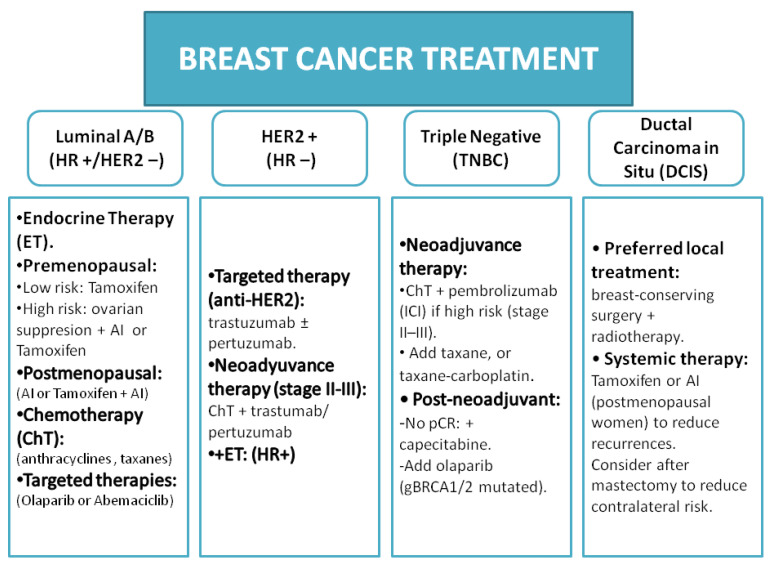
Overview of treatment schemes used in breast cancer management. This schematic summarises the standard treatment strategies for early and advanced breast cancer across molecular subtypes, highlighting that taxane-based regimens, including docetaxel, are typically integrated in neoadjuvant, adjuvant, and metastatic settings.

Breast cancer is the most frequently diagnosed malignancy in women worldwide, and it is a leading cause of cancer-related mortality. According to the most recent global estimates, breast cancer accounted for over 2.3 million new cases and more than 670,000 deaths annually in 2022 [1,2]. Chemotherapy has long been a cornerstone of treatment, particularly for aggressive and advanced breast cancers, and it remains the backbone of HER2-targeted therapies [3,4], and endocrine treatments [5] are often added. Among chemotherapeutic agents, taxanes, particularly docetaxel, play a central role in breast cancer management [6] (Figure 1). Docetaxel (Taxotere) is a highly lipophilic [7] semi-synthetic taxane that, in combination with other drugs, has significantly improved patient outcomes in both early-stage and metastatic breast cancer. For example, the addition of docetaxel to adjuvant regimens has yielded higher disease-free and overall survival rates in node-positive breast cancer patients than those for treatment using non-taxane regimens [8]. In the metastatic setting, the efficacy of docetaxel as a monotherapy was established in the pivotal 303 trial, which demonstrated superior response rates and time to progression compared to the results for doxorubicin [9]. Furthermore, docetaxel remains a key component of combination regimens, as in the CLEOPATRA trial, which showed improved outcomes with the addition of HER2-targeted agents [10]. These clinical successes have established docetaxel as a standard-of-care chemotherapeutic; it is frequently used either alone or in combination (e.g., with anthracyclines or platinum agents) for high-risk early breast cancer [8] and as a first-line treatment for HER2-positive recurrent or metastatic disease in combination with trastuzumab and pertuzumab [11]. In contrast, patients with luminal tumours are typically treated with endocrine therapy and CDK4/6 inhibitors, whereas single-agent paclitaxel is often preferred for triple-negative tumours. Combination chemotherapy, with the inclusion of docetaxel, is generally reserved for patients presenting with a high tumour burden or visceral crisis. In HER2-positive disease, the addition of docetaxel to HER2-targeted antibodies has shown synergistic effects, with the CLEOPATRA trial reporting a complete response rate of 12.7% and a partial response rate of 67.1% at week 9, which was associated with prolonged survival [10]. These outcomes were further validated in a Japanese cohort of patients with inoperable, recurrent, or advanced HER2-positive disease [12]. Despite its proven efficacy, the development of drug resistance is a major clinical challenge, which limits its long-term benefits [13,14,15,16].

Many breast tumours exhibit *de novo* (intrinsic) resistance and fail to respond to docetaxel from the outset. Even among tumours that initially respond, virtually all eventually develop acquired resistance under the selective pressure of therapy, leading to disease progression. Docetaxel resistance is associated with relapse after adjuvant chemotherapy and treatment failure in metastatic breast cancer, contributing to high mortality rates. Patients with docetaxel-resistant tumours often have limited alternative options, since cross-resistance to other chemotherapeutics is common, and resistant metastatic disease is usually incurable [15,17,18,19,20,21,22,23,24,25,26]. Docetaxel resistance is correlated with poor clinical outcomes and shorter survival [27]. Thus, understanding and overcoming resistance is critical. Elucidating the resistance mechanisms is crucial for the development of predictive biomarkers and novel therapeutic strategies to enhance patient outcomes. Table 1 summarises the predictive biomarkers of docetaxel resistance in breast cancer as obtained from recent preclinical and clinical studies.

Triple-negative breast cancer (TNBC), which lacks actionable targets, often shows poor and heterogeneous responses to taxane-based chemotherapy, partly because of the prevalence of resistance-associated biomarkers [19]. High-grade tumours may initially shrink with docetaxel treatment but then recur due to drug-resistant residual cells. Even in hormone receptor-positive cancers, docetaxel is often employed for endocrine-resistant diseases; however, cancers that have progressed despite endocrine therapy may harbour molecular alterations that confer resistance to chemotherapy [41]. Breast cancer comprises biologically distinct subtypes (luminal A/B, HER2-enriched, and triple-negative) that exhibit variable responses to docetaxel and distinct resistance mechanisms. For instance, one study found that ER-positive low-grade cell lines are more docetaxel-sensitive than are mesenchymal TNBC cell lines, which tend to exhibit higher innate resistance [22]. Clinically, this translates to variable response rates and highlights the need for biomarkers (such as tumour expression of particular genes) to predict who will benefit from docetaxel. Indeed, efforts have been made to identify such biomarkers; for example, overexpression of class III β-tubulin (encoded by *TUBB3*) or anti-apoptotic proteins in tumours has been linked to poor responses (as discussed later).

In summary, docetaxel is an important drug for breast cancer therapy, but its efficacy is curtailed by both intrinsic and acquired resistance [42]. This clinical problem has motivated extensive research on the biological underpinnings of drug resistance and potential solutions. The following sections discuss the molecular action of docetaxel, the known mechanisms by which breast cancer cells evade its effects, the special role of the actin-bundling protein fascin in tumour aggressiveness and drug resistance, an evolutionary perspective on how resistance emerges, and current advances aimed at combating docetaxel resistance.

## 2. Molecular Mechanism of Action of Taxanes (Docetaxel)

**Figure 2:**
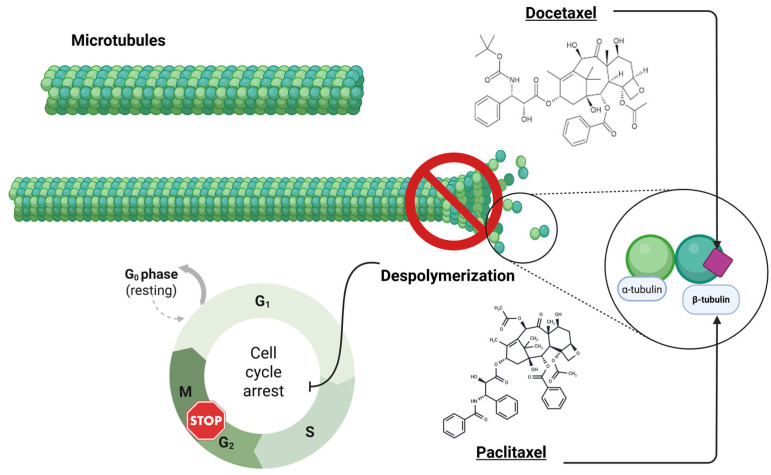
Schematic representation of the mechanisms of action of docetaxel. Docetaxel and paclitaxel bind to the β-subunit of tubulin within the microtubules, preventing depolymerisation and disrupting mitotic spindle dynamics. This leads to cell cycle arrest at the G_2_/M phase and triggers apoptosis in proliferating cancer cells.

Docetaxel is a semi-synthetic chemotherapeutic agent from the taxane family, originally derived from the *Taxus* species, that exerts its cytotoxic effect by disrupting microtubule dynamics essential for mitosis [43,44,45]. It binds specifically to a hydrophobic pocket on β-tubulin within microtubules [44], promoting tubulin polymerisation and preventing depolymerisation, thereby “locking” microtubules in a stabilised state [45]. This suppression of dynamic instability (Figure 2) impairs mitotic spindle assembly and chromosome alignment, leading to the activation of the spindle assembly checkpoint and cell cycle arrest at G2/M [46,47,48]. As a result, cells experience mitotic catastrophe and typically undergo apoptosis through the intrinsic mitochondrial pathway, which is characterised by the activation of caspase-9, caspase-3, caspase-7, and cleavage of PARP [49,50,51,52,53]. Docetaxel also induces the phosphorylation of the anti-apoptotic protein Bcl-2, thereby inhibiting its function and further promoting cell death [44]. These effects have been validated in breast cancer models, where taxane treatment induces apoptotic cascades unless counterbalanced by pro-survival signalling [22,54].

Clinically, the cytotoxic mechanism of docetaxel results in tumour shrinkage. However, the same cellular pathways that mediate its therapeutic effects, particularly apoptosis, are frequently dysregulated in cancer cells, allowing a subset of cells to evade DTX-induced death. Furthermore, the mechanism of action underlies the side effects of several drugs. For instance, peripheral neuropathy is linked to disruption of microtubule function in neuronal cells [55,56]. Therefore, a comprehensive understanding of the mode of action of docetaxel is essential for elucidating the mechanisms by which cancer cells develop resistance. Indeed, nearly all characterised resistance pathways ultimately interfere with or bypass the key effects of taxanes, including microtubule stabilisation, mitotic arrest, and the initiation of apoptosis [57].

## 3. Evolutionary Perspective: Intrinsic vs. Acquired Resistance in Cancer

Cancer can be conceptualised as an evolutionary process driven by natural selection. Tumourigenesis typically originates from a single progenitor cell that undergoes clonal expansion. However, as tumours progress, they accumulate genetic mutations, epigenetic modifications, and phenotypic variability. This intratumoural heterogeneity constitutes the substrate upon which Darwinian selection can act in combination with microenvironmental plasticity, a new concept which is part of modern extended evolutionary synthesis [58].

**Figure 3:**
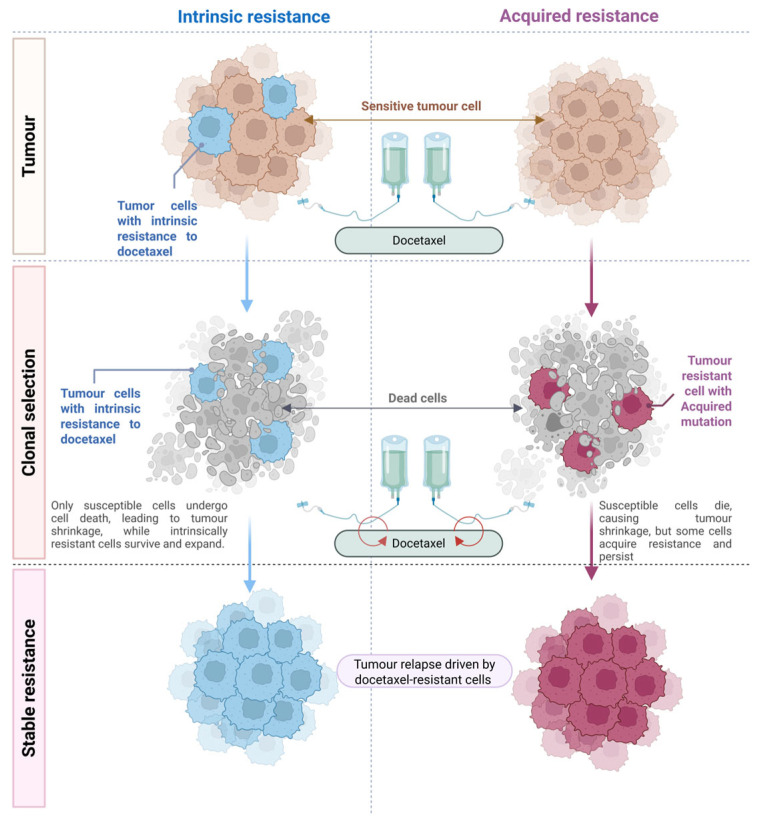
Overview of intrinsic and acquired mechanisms contributing to resistance to docetaxel. This figure illustrates how pre-existing (intrinsic) resistance and treatment-induced (acquired) adaptations enable tumour cells to survive docetaxel exposure, leading to clonal selection and tumour relapse driven by resistant subpopulations.

Exposure to chemotherapy, such as docetaxel, imposes a strong selective pressure on genetically diverse tumour populations. Sensitive cells are eliminated, while resistant subclones, whether pre-existing or adaptively induced, survive and expand, ultimately leading to treatment failure (Figure 3) [59]. This mirrors the resistance dynamics observed in infectious diseases and reflects the Darwinian model of selection within heterogeneous tumour ecosystems [59]. Intrinsic resistance arises from pre-existing traits, such as TP53 mutations, quiescent stem-like phenotypes, or high expression of efflux pumps and survival proteins (for example, PI3K/Akt activation; fascin overexpression) [60,61,62], and explains the poor initial response in some subtypes, such as TNBC [61,62].

Acquired resistance develops during therapy through selection of minor resistant subclones or via adaptive rewiring (e.g., β-tubulin mutations and ABC transporter upregulation) [63,64,65]. Genomic instability in cancer cells facilitates multiple escape routes; for example, PIK3CA activation or new TP53 mutations can emerge during treatment [53]. These traits may incur fitness costs; therefore, intermittent therapy (“chemo holidays”) can allow sensitive cells to regain dominance, a concept explored in adaptive therapy [59]. Clonal shifts observed through serial biopsies or ctDNAs support this dynamic [66].

Resistance mechanisms often exhibit pathway-plasticity. Blocking one survival route can activate compensatory signalling (for example, PI3K–MAPK) [67]. Thus, tumours behave as evolving ecosystems, with interclonal competition shaped by selective pressure and microenvironmental constraints [68,69]. Docetaxel-resistant clones frequently display mesenchymal traits, high efflux transporter expression, or quiescence [70], and these features may be intrinsically present or acquired upon exposure. Overall, resistance should be viewed not as binary but as a shifting evolutionary continuum.

In summary, intrinsic resistance represents a pre-existing escape mechanism, whereas acquired resistance reflects an adaptive response that evolves during treatment. Both arise through evolutionary selection processes. Recognising this evolutionary underpinning enables researchers and clinicians to better anticipate therapeutic resistance. It must be assumed that given sufficient time and selective pressure, cancer will evolve mechanisms to evade treatment [71]. Therefore, the clinical objective is to either delay the emergence of resistance or to effectively target resistant clones once they appear. In the following section, we review the molecular mechanisms by which breast cancer cells acquire resistance to docetaxel, many of which may operate in both intrinsic and acquired contexts.

## 4. Mechanisms of Docetaxel Resistance in Breast Cancer

**Figure 4:**
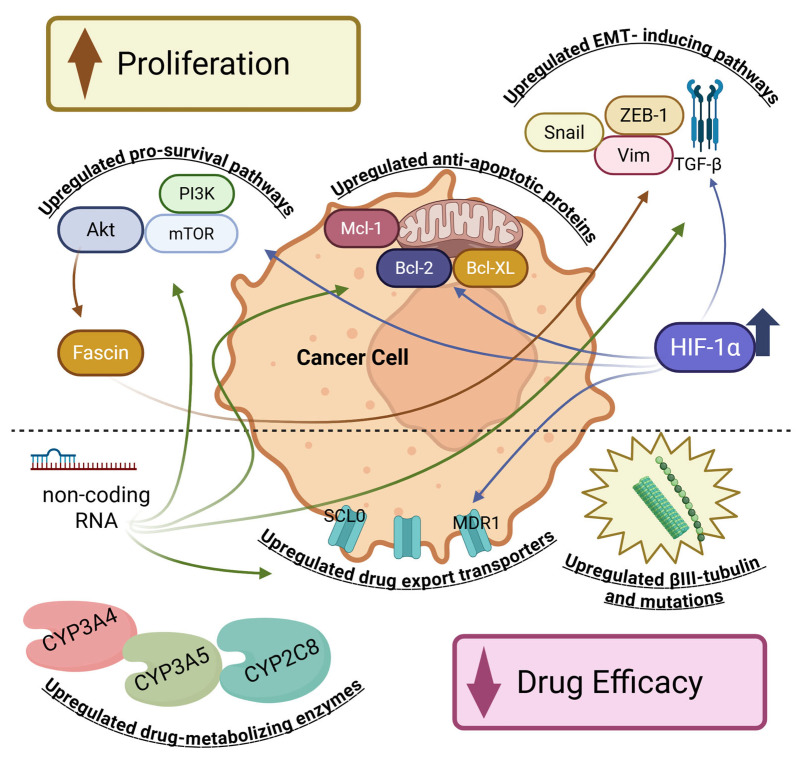
Schematic representation of the mechanisms underlying resistance to docetaxel. Mechanisms that enhance cell proliferation, depicted at the top of the image, include increased expression of anti-apoptotic genes and overactivation of survival pathways like PI3K/AKT. Conversely, resistance mechanisms aimed at reducing drug efficacy are shown at the bottom, including the upregulation of efflux transporters such as MDR, βIII-tubulin, metabolic enzymes, and non-coding RNAs.

Breast cancer cells employ a variety of mechanisms to evade the cytotoxic effects of docetaxel. These mechanisms can be broadly categorised into several groups, often overlapping and co-occurring within the same tumour. Key resistance mechanisms include (1) alterations in the target of the drug (microtubules), (2) increased drug efflux and decreased drug accumulation, (3) evasion of apoptosis and cell death, (4) activation of alternative survival signalling pathways and epithelial-to-mesenchymal transition (EMT), and (5) cancer stem cell features and tumour microenvironment influences (Figure 4).

### 4.1. Alterations in Tubulin and Microtubule Dynamics

Taxanes, such as docetaxel, stabilise microtubules by binding to β-tubulin, preventing depolymerisation and inducing mitotic arrest. Resistance frequently arises from alterations in tubulin composition or structure that impair drug binding [19,71,72]. Overexpression of class III β-tubulin (TUBB3) has been consistently linked to taxane resistance in several cancers, including breast cancer [73,74]. Resistant cells often exhibit higher TUBB3 levels and elevated docetaxel IC_50_ values; for instance, MDA-MB-231 cells express four-fold more TUBB3 than luminal MCF7 cells [22,73]. Additionally, point mutations in β-tubulin (for example, A185T, A248V, and R306C) may disrupt taxane–tubulin interactions without abolishing microtubule function [71,72,73].

Post-translational modifications (PTMs) such as tubulin acetylation or detyrosination can stabilise microtubules independently of taxanes and reduce drug efficacy. Similarly, microtubule-associated proteins (MAPs) such as Tau, MAP4, and stathmin modulate polymer dynamics and influence the taxane response. High Tau expression has been associated with resistance, particularly in ER-positive breast tumours [75,76,77,78], whereas stathmin overexpression enhances microtubule turnover and opposes taxane activity [79,80].

Chronic exposure to docetaxel may also lead to adaptive changes in spindle architecture and microtubule behaviour that enable resistant cells to bypass mitotic arrest [39,81]. These multifactorial alterations, including isotype switching, PTMs, and MAP deregulation, converge to reduce the cytotoxic effects of docetaxel.

### 4.2. Increased Drug Efflux and Decreased Intracellular Drug Accumulation

One of the most well-characterised mechanisms of docetaxel resistance is the active efflux of cytotoxic drugs via ATP-binding cassette (ABC) transporters [82]. Among them, P-glycoprotein (P-gp), encoded by ABCB1/MDR1, has been the most studied. Its overexpression reduces intracellular docetaxel levels, thereby limiting cytotoxic efficacy. In breast cancer, ABCB1 expression may be intrinsically elevated, particularly in aggressive subtypes such as TNBC, or induced by chemotherapeutic pressure [15,83].

Functional inhibition or genetic silencing of P-gp restores docetaxel sensitivity in resistant cell lines [83]. Bufalin, a bioactive compound used in traditional Chinese medicine, downregulates ABCB1 protein levels and ATPase activity, enhancing docetaxel retention and apoptosis in vitro and reducing tumour growth in xenograft models [83]. Clinically, elevated ABCB1 levels in tumour specimens correlate with a reduced taxane response and earlier relapse [83].

In addition to P-gp, other ABC transporters such as ABCC1 (MRP1), ABCC10 (MRP7), and ABCG2 (BCRP) also mediate taxane efflux [29,84,85]. Notably, ABCC10 overexpression decreases taxane sensitivity in breast cancer, whereas genetic ablation increases susceptibility [84,85]. Although hepatic CYP3A4 enzymes contribute to taxane metabolism, they play only a minor role in efflux-mediated resistance [86].

Several pharmacological inhibitors of P-gp have been tested, including verapamil [87] and more specific MDR1 antagonists [88]; however, their clinical application is limited by poor selectivity and systemic toxicity [89]. To overcome these limitations, new approaches include nanotechnological delivery platforms designed to bypass efflux, such as albumin-bound paclitaxel (nab-paclitaxel) and liposomal or micellar formulations of docetaxel [90,91,92].

In summary, multidrug efflux transporters, particularly P-gp, constitute a central mechanism of docetaxel resistance in breast cancer. Their assessment may inform treatment decisions, whereas pharmacological inhibition and drug delivery innovations hold promise for improving taxane efficacy.

### 4.3. Evasion of Apoptosis and Cell Death Programs

Docetaxel induces apoptosis, primarily via the intrinsic (mitochondrial) pathway through the activation of caspase-9, caspase-3/7, and cleavage of PARP [49,50]. However, chemoresistant breast cancer cells evade apoptosis by altering multiple molecular checkpoints. A key mechanism involves overexpression of anti-apoptotic proteins, such as BCL-2, BCL-xL, MCL-1, and IAP family members like XIAP and survivin, which inhibit caspase activation and mitochondrial membrane permeabilisation [73,93,94,95,96]. The co-expression of survivin and TUBB3 has been associated with a reduced response to docetaxel [73], and XIAP or Livin is upregulated in fascin-positive, chemoresistant cells [62,93].

Apoptosis can also be suppressed by the inactivation of pro-apoptotic factors. TP53 mutations, which are frequent in triple-negative and HER2-positive breast cancers, reduce apoptotic priming and contribute to resistance, despite taxanes being partly effective in a p53-independent manner [94,95,97]. Downregulation of BAX, BAK, and executioner caspases also impair cell death. Moreover, fascin can promote resistance by activating the PI3K/Akt and NF-κB pathways, which suppresses pro-apoptotic proteins and increases XIAP expression [49]. These pathways are often constitutively active in resistant cells, shifting the balance towards survival [93].

Mitotic slippage is another evasion mechanism by which cells escape prolonged mitotic arrest without dying, resulting in tetraploidy or senescence [49,71,98,99]. This process is facilitated by weakened spindle checkpoint signalling and the concurrent activation of survival pathways.

Clinically, tumours with high BCL-2 expression show poor pathological responses to chemotherapy, and combinations of docetaxel with BCL-2 inhibitors, such as venetoclax, are under investigation in triple-negative breast cancer [96,97]. These findings support the rational combination of cytotoxic agents with drugs targeting apoptotic checkpoints to restore cell death and overcome resistance.

### 4.4. Activation of Survival Pathways and EMT

Under therapeutic pressure, cancer cells activate prosurvival and developmental pathways that sustain growth and confer chemoresistance. In breast cancer, several interconnected cascades contribute to docetaxel resistance.

The PI3K/Akt pathway is one of the most frequently upregulated genes driven by mechanisms such as PTEN loss, PIK3CA mutations, and growth factor receptor activation (for example, IGF-1R; HER2) [22,100,101,102]. Akt promotes cell survival and cell cycle progression, and its phosphorylation is elevated in docetaxel-resistant breast cancer cells. In tamoxifen-resistant MCF7 models, increased Akt activity correlates with enhanced docetaxel tolerance [23,89]. Similarly, the RAS–RAF–MEK–ERK (MAPK) cascade promotes proliferation and has been linked to taxane resistance, particularly in tumours with HER2 overexpression or RAS activation. EGF stimulation of MAPK signalling induces EMT and fascin expression, thereby enhancing migration and resistance [103,104].

NF-κB, a transcription factor that regulates survival and inflammation, is constitutively active in DTX-resistant breast cancer cells. It promotes resistance by upregulating anti-apoptotic proteins (BCL-xL and XIAP) and EMT-related effectors (MMP-2, MMP-9, and uPA) [105,106]. Fascin activates NF-κB, forming a positive feedback loop that enhances cell survival and invasiveness [103,105]. These signals may be triggered by cytokines from the tumour microenvironment (for example, IL-6) or via IKK-mediated intracellular activation, linking extrinsic and intrinsic resistance mechanisms. Notably, pharmacological inhibition of NF-κB using compounds such as SN-50 or baicalin restores docetaxel sensitivity in resistant breast cancer models, both in vitro and in vivo [107,108]. These findings underscore the potential of NF-κB as a therapeutic target for overcoming chemoresistance.

EMT imparts intrinsic resistance by promoting a mesenchymal, stem cell-like phenotype associated with slow proliferation, ABC transporter upregulation, and anti-apoptotic gene expression [109,110,111]. EMT markers include E-cadherin loss and upregulation of vimentin, N-cadherin, ZEB1/2, and Snail and Twist [110]. The induction of EMT by TGF-β or EMT-TFs reduces docetaxel sensitivity, whereas EMT reversal restores chemosensitivity [111,112,113].

Non-coding RNAs modulate EMT and resistance. In lung cancer, lncRNA linc-ROR sponges miR-145, derepressing fascin and promoting EMT and resistance, which is reversible upon knockdown [114]. In breast cancer, the loss of miR-200c allows ZEB1-driven EMT and chemoresistance, while restoring miR-200c sensitises cells to docetaxel [115,116]. Additionally, miR-141 has been implicated in resistance via the regulation of EIF4E and EMT/MAPK signalling [117].

The Notch pathway promotes EMT and cancer stemness. Inhibition of Notch1/4 using γ-secretase inhibitors (e.g., PF-03084014) reverses EMT; downregulates Snail, Slug, and ABCB1; and enhances docetaxel-induced apoptosis in TNBC models [110]. Notch also induces ZEB1, N-cadherin, and fascin expression, and evidence from pancreatic cancer suggests that a Notch → Akt → fascin axis likely operates in breast cancer [118].

In summary, docetaxel-resistant breast cancer cells activate the pro-survival (PI3K/Akt, MAPK, NF-κB, and Notch) and EMT pathways to bypass cytotoxic stress. EMT imparts stemness, drug efflux, and apoptotic resistance, whereas fascin amplifies these phenotypes. These findings support combination therapies targeting both taxane vulnerability and resistance networks, such as AKT inhibitors (ipatasertib) and mTOR inhibitors (everolimus), especially in tumours with PIK3CA mutations.

### 4.5. Cancer Stem Cells and Tumour Heterogeneity

Cancer stem cells (CSCs) constitute a subpopulation of breast tumours that are characterised by self-renewal, quiescence, and resistance to therapy. They are commonly identified by the CD44^+^/CD24^−^ phenotype and high ALDH activity, and are typically spared by chemotherapy, allowing tumour repopulation [119]. Exposure to docetaxel can enrich CSC-like cells, consistent with selective survival under cytotoxic pressure [119].

Fascin (FSCN1), which is markedly upregulated in triple-negative breast cancer (TNBC) [120], promotes CSC traits by activating the Notch and β-catenin pathways [119,121]. Fascin overexpression enhances mammosphere formation and resistance, while its silencing reduces the CD44^+^/CD24^−^ fraction, impairs stem-like features, and sensitises tumours to docetaxel [119,122]. These findings highlight fascin as a convergence point linking EMT, stemness, and resistance.

The tumour microenvironment (TME) further contributes to docetaxel resistance. Hypoxic niches promote EMT, slow proliferation, and induce HIF-1α, which upregulates both MDR1 and FSCN1 [103,123,124,125]. Stromal components, such as cancer-associated fibroblasts and tumour-associated macrophages, secrete IL-6 and activate the STAT3 and NF-κB pathways in cancer cells [105,126]. IL-6/STAT3 signalling has also been shown to increase fascin expression, linking microenvironmental cues to intrinsic resistance mechanisms [103,127].

In summary, docetaxel resistance in breast cancer arises not only from tumour-intrinsic changes, but also from CSC enrichment and TME-mediated adaptation. Therapeutic strategies targeting CSCs (e.g., Notch or Wnt inhibitors) or modulation of the TME (for example, IL-6 or HIF-1α blockade) holds promise for enhancing the efficacy of taxane-based regimens.

## 5. Role of Fascin in Tumour Biology and Chemoresistance

Fascin, encoded by *FSCN1*, is an actin-bundling protein primarily expressed in dendritic cells under physiological conditions. In cancer, fascin is frequently upregulated and contributes to cellular motility and invasion by organising actin into tightly packed filopodia and invadopodia [62,128]. In breast cancer, high fascin expression correlates with aggressive histological features, triple-negative subtype, early metastasis, and reduced overall survival. Ghebeh et al. first reported that high fascin expression confers chemoresistance in breast cancer [62]. Fascin-positive tumours exhibited a poorer response to chemotherapy and reduced apoptosis compared to the results for their fascin-negative counterparts. Mechanistically, fascin enhanced PI3K/Akt activation and upregulated anti-apoptotic proteins such as XIAP and Livin, while reducing the levels of active caspase-3/9 and cleaved PARP, thereby suppressing apoptotic responses to docetaxel. A recent study in colorectal cancer demonstrated that a variant in the lncRNA CCAT1 (rs67085638) promotes paclitaxel resistance via miR-24-3p-mediated upregulation of fascin, which activates the PI3K/Akt pathway and suppresses apoptosis [129]. Fascin also modulates focal adhesion kinase (FAK) and NF-κB signalling, which promotes survival and invasive behaviour. In fascin-positive cells, increased FAK phosphorylation has been linked to downstream NF-κB activation and the transcription of pro-survival genes [122]. Moreover, fascin reinforces CSC maintenance through the Notch–integrin β1 axis and supports EMT phenotypes, contributing to both migratory potential and drug tolerance [119,121]. In docetaxel-resistant models of lung cancer, fascin was upregulated and mediated resistance via EMT; silencing fascin or restoring miR-145 expression re-sensitised cells to treatment [121]. Notably, fascin-driven resistance extends beyond breast cancer [130,131]. In cervical, prostate, and lung cancers, fascin expression has been associated with resistance to cisplatin, paclitaxel, and docetaxel, often through EMT-linked mechanisms or miRNA dysregulation [114,132,133].

Given its virtually absent expression in normal epithelia, fascin is a promising ther-apeutic target. Small-molecule fascin inhibitors such as the NP-G2-029/NP-G2-044 series have demonstrably reduced breast cancer cell invasion and metastasis, showing syner-gistic effects with paclitaxel-based chemotherapy in murine models [134,135,136]. Whether such compounds can reverse chemoresistance remains unclear.

Clinically, assessing fascin expression may offer prognostic value and help to stratify patients. Tumours with high fascin expression could benefit from more aggressive upfront therapy or inclusion in trials evaluating fascin inhibitors (for example, NP-G2-044), or PI3K/Akt pathway inhibitors. Two ongoing clinical trials (Clinical Trials: NCT05023486; EudraCT: 2021-001328-17), in which fascin inhibitors are being tested to treat different tumours, including breast cancer, will provide novel insights about mechanisms of reverting chemoresistance [135].

In summary, fascin integrates key hallmarks of cancer aggressiveness, including motility, stemness, survival, and drug resistance. Targeting this multifunctional protein may not only inhibit metastatic progression but also restore chemosensitivity, representing a dual-pronged therapeutic approach for breast cancer.

## 6. Current Research Advances and Future Directions

Research over the last several years has focused on unravelling the complexities of docetaxel resistance and finding ways to overcome it. Some recent advances and emerging insights include the following.

### 6.1. Genomic and Transcriptomic Profiling of Resistant Tumours

Modern sequencing techniques are applied to patients before and after chemotherapy to identify key genetic changes associated with resistance. These studies have reinforced the role of known players (e.g., *TP53* mutations, PIK3CA pathway alterations, and MDR1 upregulation) and uncovered new candidates. For instance, 2023 integrative analyses identified a set of differentially expressed genes and non-coding RNAs in residual disease after neoadjuvant taxane therapy, suggesting new biomarkers of resistance [137]. There is also an interest in polyclonal resistance, in which different metastases or regions of a tumour employ distinct resistance mechanisms, complicating treatment.

### 6.2. Non-Coding RNAs (lncRNAs, miRNAs, and circRNAs)

The role of ncRNAs in chemoresistance has come to the forefront. Long non-coding RNAs (lncRNAs) can regulate gene expression at multiple levels, and have been implicated in modulating drug responses. A comprehensive 2023 review catalogued numerous lncRNAs involved in taxane resistance in breast cancer [44]. For example, LINC00680 was found to be upregulated in docetaxel-resistant breast cancer cells and promotes resistance by sponging miR-432 to upregulate TRIM14 (which affects NF-κB signalling)—this was noted as one mechanism by which lncRNAs contribute to taxane resistance [137,138]. Another lncRNA, ROR, connects to EMT and fascin via miR-145 sponging (although shown in lung cancer, similar principles apply) [114]. The loss of tumour-suppressive miRNAs (such as the miR-200 family or miR-145) or overexpression of oncogenic miRNAs (such as miR-21) has been associated with taxane resistance phenotypes. Restoration of certain miRNAs in cell models can re-sensitise cells to docetaxel by targeting the mRNAs of resistance-related genes.

Circular RNAs (circRNAs) are a novel class of non-coding RNA that have gained increasing attention. These covalently closed RNA loops often function as miRNA sponges. Recent studies have identified specific circRNAs that confer docetaxel resistance. For example, circABCB1, derived from the ABCB1 gene, has been shown to diminish sensitivity to docetaxel in breast cancer, presumably by sponging miRNAs that would normally downregulate ABCB1, thereby indirectly maintaining high P-gp levels [33]. Another circUBR5 was recently reported (2024) to promote ribosome biogenesis and docetaxel resistance in triple-negative breast cancer via the miR-340-5p/CMTM6/c-MYC axis [34]. In that study, circUBR5 acted as a sponge for miR-340-5p, leading to increased expression of CMTM6 and c-MYC, which drove protein synthesis and cell growth, ultimately rendering cells less susceptible to docetaxel-induced stress (faster proliferation can antagonise the cytostatic effect of the drug). Targeting circRNAs is still in its early stages, but these findings open up new potential molecular targets for reversing resistance.

### 6.3. Novel Therapeutic Agents and Combinations

There is ongoing research on drugs that can overcome the known resistance mechanisms or kill cells via alternative lethal pathways. Table 2, Table 3, Table 4, Table 5 and Table 6 summarise clinical trials incorporating docetaxel as part of the therapeutic intervention in breast cancer. Some avenues to explore include the following:

- Next-generation taxanes: Analogues such as cabazitaxel (a semisynthetic taxane) have been developed to evade P-gp efflux and exhibit activity in docetaxel-resistant tumours (cabazitaxel is approved in prostate cancer after docetaxel failure). Trials on breast cancer are not ongoing, but cabazitaxel and other drugs (e.g., larotaxel) may offer options for taxane-resistant cases [139,140,141].
- Tubulin inhibitors with different mechanisms: Epothilones (such as ixabepilone) also stabilise microtubules but can retain activity in some taxane-resistant tumours, particularly those overexpressing TUBB3, because epothilones bind β-tubulin at different sites. Ixabepilone has been tested in breast cancer and approved for use in certain resistant metastatic cases, highlighting how drugs in the same functional class can sometimes overcome specific resistance, such as P-gp (ixabepilone is a poor P-gp substrate) or β-tubulin alterations [142,143].
- Targeted pathway inhibitors: Combining docetaxel with targeted inhibitors is a major research area. PI3K inhibitors (e.g., buparlisib and alpelisib) and AKT inhibitors have been used to overcome resistance mediated by the PI3K/Akt pathway [144]. mTOR inhibitors (everolimus) combined with taxanes have shown some synergy in preclinical models, and a clinical trial of everolimus with weekly paclitaxel showed improved pathological complete response in HER2-negative breast cancer, suggesting that a similar strategy might improve docetaxel efficacy [145]. Notch pathway inhibitors (gamma secretase inhibitors) and hedgehog inhibitors are being studied to deplete cancer stem cells and potentially improve the chemo response [146,147]. There is also interest in inhibiting anti-apoptotic proteins; for example, a phase I trial combining venetoclax (BCL-2 inhibitor) with pegylated liposomal doxorubicin in TNBC could be envisioned with docetaxel, if the preclinical rationale is strong.
- Immunotherapy combinations: While not directly reversing a resistance mechanism in the classical sense, the use of immunotherapy (such as checkpoint inhibitors) with chemotherapy can provide an alternative way to kill tumour cells by harnessing the immune system. In TNBC, the addition of atezolizumab (anti-PD-L1) to nab-paclitaxel improves outcomes in PD-L1+ patients. Trials have added checkpoint inhibitors to docetaxel. The idea is that even if some cells resist the direct effect of docetaxel, they may become more immunogenic (chemotherapy can cause immunogenic cell death in some cases), and immunotherapy can then eliminate those cells. In addition, chemotherapy may modulate the immune environment to make immunotherapy more effective. This multipronged kill approach may circumvent individual cellular resistance mechanisms.

**Table 2:** Clinical trials in early breast cancer HR+ Her2—recruiting, active, not recruiting—with docetaxel interventions.

NTC Registry	Study Title	Phase	Interventions	Design	Start Date	References
NCT05165225	Phase II Neoadjuvant Pyrotinib Combined with Neoadjuvant Chemotherapy in HER2-Low-Expressing and HR Positive Early or Locally Advanced Breast Cancer: a Single-Arm, Non-Randomized, Single-Center, Open Label Trial	2	pyrotinib + epirubicin and cyclophosphamide followed by docetaxel	NA; single group	13 July 2021	[148]
NCT04293393	Neoadjuvant Study Chemotherapy vs. Letrozole + Abemaciclib in HR+/HER2− High/Intermediate Risk Breast Cancer Patients	2	doxorubicin + cyclophosphamide + docetaxel vs. letrozole +abermaciclib +/− LHRH	RCT; open label	1 October 2020	[149]
NCT03201861	Addition of Cisplatin to Adjuvant Chemotherapy for Early-Stage Breast Cancer in High-Risk Women	3	wpirubicin + cyclophospamide to docetaxel or paclitaxel vs. paclitaxel + cisplatin	RCT; open label	27 July 2017	[150]
NCT06107673	Dalpiciclib Plus AI (Neoadjuvant Endocrine Therapy) Compared with Neoadjuvant Chemotherapy in Early Breast Cancer (EBC)	2	NACT-dalpiciclib vs. ciclophosphamide + docetaxel	RCT; masking triple	30 September 2023	[151]

NACT: neoadjuvant chemotherapy; RCT: randomized controlled trial; NA: no allocation.

**Table 3:** Clinical trials in breast cancer HR+ Her2—recruiting, active, not recruiting—with docetaxel interventions.

NTC Registry	Study Title	Phase	Interventions	Design	Start Date	References
NCT06009627	Study of Neoadjuvant Endocrine Therapy in HR Positive and HER2 Negative Premenopausal Breast Cancer Patients	2/3	darxil + exenestane + goserelin vs. docetaxel + doxorubicin + cyclophosphamide	R; open label	11 April 2023	[152]
NCT03701334	A Trial to Evaluate Efficacy and Safety of Ribociclib with Endocrine Therapy as Adjuvant Treatment in Patients With HR+/HER2- Early Breast Cancer (NATALEE)	3	ribociclib + endocrine therapy (ET) vs. ET	RCT; open label	10 October 2018	[153]
NCT06375707	Efficacy and Safety of Ribociclib in Combination with NSAI vs. Physician’s Choice of Chemotherapy Sequential Endocrine Therapy in HR+/HER2− Advanced Breast Cancer	2	docetaxel or paclitaxel + vinorelbine + capecitabine: sequential ribociclib: 600mg/d, 3 weeks continuous oral withdrawal for 1-week NSAI: anastrozole 1mg, 1 time/d, oral or letrozole: 2.5mg, 1 time/d, oral	RCT; open label	9 January 2024	N/A
NCT04872985	Pyrotinib in Combination with Neoadjuvant Chemotherapy in HR+/HER2-, HER4 High Expression Breast Cancer Patients: a Phase II Trial	2	pyrotinib + doxorubicin/epirubicin + cyclophosphamide followed by docetaxel/nab-paclitaxel	RCT; DB	20 April 2021	[148]
NCT05296746	Neoadjuvant and Adjuvant Ribociclib and ET for Clinically High-Risk ER+ and HER2- Breast Cancer	2	NACT–ACT ribociclib + letrozole (responder) vs. (non-responder NACT: ribociclib + letrozole ACT—Arm1: doxorubicin + cyclophosphamide + docetaxel; Arm2: docetaxel + cyclophosphamide; Arm3: paclitaxel + doxorubicin + cyclophosphamide. All patients receive ribociclib + letrozole or AI	NR; parallel assigned	3 May 2022	[154]

DB: double-blind; NACT: neoadjuvant chemotherapy; ACT: adjuvant chemotherapy; NR: non-randomized; RCT: randomized controlled trial.

**Table 4:** Clinical trials in breast cancer HR+/- Her2+—recruiting, active, not recruiting—with docetaxel interventions.

NTC Registry	Study Title	Phase	Interventions	Design	Start Date	References
NCT05638594	Pyrotinib Combined with Trastuzumab, Dalpiciclib, Letrozole vs. TCbHP (Trastuzumab Plus Pertuzumab with Docetaxel and Carboplatin) as Neoadjuvant Treatment in HR +/HER2 + Breast Cancer	2	pyrotinib + trastuzumab + dalpiciclib + letrozole vs. trastuzumab + pertuzumab + docetaxel + carboplatin	RCT; open label	20 December 2022	[155]
NCT05346224	A Study to Evaluate the Efficacy and Safety of HLX11 vs. EU-Perjeta^®^ in the Neoadjuvant Therapy of HER2-Positive and HR-Negative Early-Stage or Locally Advanced Breast Cancer	3	NACT: HLX11 + trastuzumab docetaxel; ACT: doxorubicin + cyclophosphamide + trastuzumab +HLX11 vs. NACT: EU-Perjeta^®^ + trastuzumab docetaxel; ACT: doxorubicin + cyclophosphamide + trastuzumab + EU-Perjeta^®^	RCT; open label	17 April 2022	[156]
NCT05319873	Ribociclib, Tucatinib, and Trastuzumab for the Treatment of HER2 Positive Breast Cancer	1/2	Phase 1b; ribociclib + tucatinib + trastuzumab, if no progression diseases or unacceptable toxicity, allowed to Phase 2 Arm A: ribociclib + tucatinib + trastuzumab + fulvestrant; Arm B: docetaxel + carboplatin + trastuzumab; Arm C: ribociclib + tucatinib + trastuzumab	RCT; sequential assignment	7 April 2022	[157]
NCT05900206	Trastuzumab Deruxtecan vs. Standard Neoadjuvant Treatment for HER2-Positive Breast Cancer	2	Arm 1: trastuzumab deruxtecan; Arm 2: docetaxel/paclitaxel + carboplatin + trastuzumab + pertuzumab; Arm 3: (ER + and luminal) ribociclib + letrozole; Arm 4: (ER- and luminal) epirubicin vs. cyclosporine Arm 5: trastuzumab deruxtecan or docetaxel/paclitaxel + carboplatin + trastuzumab + pertuzumab	RCT; parallel assigment; open label	26 October 2023	Link
NCT06770296	The Dosing Regimen of Pyrotinib in HER2-Positive Advanced First-Line Breast Cancer: a Phase I Clinical Study	1	pyrotinib low dose + trastuzumab + docetaxel vs. pyrotinib normal dose + trastuzumab + docetaxel	RCT; sequential assignment; open label	1 November 2024	N/A
NCT05704829	NeoAdjuvant Therapy With Trastuzumab-deruxtecan vs. Chemotherapy+Trastuzumab+Pertuzumab in HER2+ Early Breast Cancer	2	T-DXd iv NACT-ACT vs. pacli-/docetacel + carboplatin + trastuzumab + pertuzumab	RCT; crossover assignment; open label	5 February 2024	[158]
NCT05720026	Study to Evaluate the Efficacy and Safety of SYSA1901 vs. Perjeta^®^ of HER2-Positive Breast Cancer	3	SYSA1901 + trastuzumab + docetaxel vs. Perjeta^®^ + trastuzumab + docetaxel	RCT; DB; parallel-controlled	9 January 2023	N/A
NCT06278870	Disitamab Vedotin + Pyrotinib vs. THP in the First-Line Treatment for HER2+ Advanced Breast Cancer Clinical Trial	3	disitamab vedotin + pyrotinib + trastuzumab vs. trastuzumab + pertuzumab + docetaxel/paclitaxel/paclitaxel liposomal/paclitaxel alb.	RCT; quadruple; parallel assignment	30 June 2023	[159]
NCT06747338	A Phase III Study of KN026 in Combination with HB1801 ± Carboplatin as Neoadjuvant Treatment for Early or Locally Advanced HER2-Positive Breast Cancer	3	KN026 + HB1801 + carboplatin vs. trastuzumab + pertuzumab + docetaxel + carboplatin	RCT; parallel assignment; open label	16 December 2024	[160]
NCT06038539	Efficacy and Safety of the Proposed Biosimilar Pertuzumab (PERT-IJS) vs. EU-Perjeta^®^ Along with Trastuzumab and Chemotherapy (Carboplatin and Docetaxel) as Neoadjuvant Treatment in Chemotherapy naïve Patients with Early Stage or Locally Advanced HR Negative and HER2 Positive Breast Cancer	3	PERT-IJS + trastuzumab + carboplatin and docetaxel vs. EU-Perjeta^®^ + trastuzumab + carboplatin and docetaxel	RCT; DB; parallel assignment	31 January 2025	[161]

DB: double-blind; RCT: randomized controlled trial; NACT: neoadjuvant chemotherapy; ACT: adjuvant chemotherapy.

**Table 5:** Clinical trials in metastatic breast cancer HR+/- Her2+—recruiting, active, not recruiting—with docetaxel interventions.

NTC Registry	Study Title	Phase	Interventions	Design	Start Date	References
NCT04760431	TKIs vs. Pertuzumab in HER2+ Breast Cancer Patients with Active Brain Metastases (HER2BRAIN)	2	trastuzumab docetaxel pyrotinib vs. trastuzumab + docetaxel + pertuzumab	RCT; 1:1	25 January 2021	N/A
NCT05621434	A Study to Evaluate Inetetamab + Pyrotinib + Chemotherapy in Previously Untreated HER2-Positive Metastatic Breast Cancer	2	inetetamab + pyrotinib and (taxane, vinorelbine, capecitabine, eribulin, and other agents (physicians choices).	NR	10 December 2022	[162]
NCT05296798	A Study to Evaluate the Efficacy and Safety of Giredestrant in Combination with Phesgo (Pertuzumab, Trastuzumab, and Hyaluronidase-zzxf) vs. Phesgo in Participants with Locally Advanced or Metastatic Breast Cancer (heredERA Breast Cancer)	3	Induction: giredestrant + pertuzumab + trastuzumab + taxane. Maintenance Arm A: giredestrant + pertuzumab + trastuzumab or Arm B: giredestrant + pertuzumab + trastuzumab + ET	RCT; open label	18 July 2022	[163]
NCT06057610	A Phase III Study of SHR-A1811 Injection with or Without Pertuzumab in HER2-Positive Recurrent or Metastatic Breast Cancer	3	A: SHR-A1811; B: SHR-A1811 + pertuzumab; C: trastuzumab + pertuzumab + docetaxel	RCT; open label	16 October 2023	[164]
NCT05698186	Thero2-01S22 in HER2-Positive Breast Cancer	3	thero2-01S22/placebo + docetaxel or vinorelbine + pertuzumab + trastuzumab	RCT; DB; placebo-controlled	15 May 2023	N/A
NCT06135714	Metastasis-Directed Therapy for Oligometastases of Breast Cancer	3	Luminal BC: CDK4/6 inhibitors + ET; HER2+: trastuzumab + pertuzumab + docetaxel; TNBC: immune checkpoint inhibitors expressing PD-L1; Arm A continues systemic chemotherapy alone; Arm B followed the same treatment.	RCT; parallel assignment; open label	11 August 2023	[165]
NCT06439693	The SAPPHO Study: Sequential Therapy with Curative Intent in de Novo HER2+ Metastatic Breast Cancer	2	taxane + trastuzumab + pertuzumab followed by trastuzumab deruxtecan, followed by tucatinib + ado-trastuzumab emtansine, followed by trastuzumab + pertuzumab + tucatinib	NA; single group sequential treatment	8 August 2024	N/A
NCT06445400	A Study of BL-M07D1, BL-M07D1+Pertuzumab and BL-M07D1+Pertuzumab+Docetaxel in Patients with Unresectable Locally Advanced or Metastatic HER2-Positive Breast Cancer	2	Arm A: BL-M07D1 + pertuzumab; Arm B: BL-M07D1 + pertuzumab + docetaxel	NA; single group; open label	19 June 2024	N/A
NCT07003074	A Clinical Study of TQB2102 vs. Docetaxel Plus Trastuzumab and Pertuzumab in the Treatment of HER2 Positive Recurrent or Metastatic Breast Cancer	3	Arm A: TQB2102; Arm B: docetaxel + trastuzumab + pertuzumab	RCT; open label; parallel-controlled	August 2025	[166]

ET: endocrine therapy; DB: double-blind; NR: non-randomized; RCT: randomized controlled trial; NA: no allocation.

**Table 6:** Clinical trials in triple negative breast cancer (TNBC)—recruiting, active, not recruiting—with docetaxel interventions.

NTC Registry	Study Title	Phase	Interventions	Design	Start Date	References
NCT04836156	Neoadjuvant Therapy Study Guided by Drug Screening in Vitro for Human Epidermal Growth Factor Receptor 2 (HER2) Negative Early Breast Cancer Patients	1/2	docetaxel + carboplatin docetaxel + epirubicin	NR; single group assignment; open label	2 April 2021	[167]
NCT05475678	Clinical Study of Camrelizumab Combined with TCb vs. TCb in Neoadjuvant Treatment of Triple-Negative Breast Cancer	2	carrelizumab + docetaxel + carboplatin vs. docetaxel + carboplatin	RCT; parallel assignment; open label	19 July 2022	[168]
NCT05645380	Neoadjuvant TIL- and Response-Adapted Chemoimmunotherapy for TNBC	2	Arm A: carboplatin + docetaxel + pembrolizumab; Arm B: carboplatin + docetaxel + doxorubicin + cyclophosphamide + pembrolizumab	NR; parallel assignment; open label	5 December 2022	[169]
NCT04947189	Seviteronel in Combination with Chemotherapy in Androgen-receptor Positive Metastatic Triple-Negative Breast Cancer	1/2	seviteronel + dexamethasone + docetaxel	NR; single group; open label	1 November 2021	[170]
NCT05076760	MEM-288 Oncolytic Virus Alone and in Combination with Standard of Care Therapy in Advanced Solid Tumours	1	MEM-288 vs. nivolumab + docetaxel	NR; single group; open label	21 April 2022	[171]
NCT05929768	Shorter Anthracycline-Free Chemo Immunotherapy Adapted to Pathological Response in Early Triple Negative Breast Cancer (SCARLET), A Randomized Phase III Study	3	paclitaxel + carboplatin + pembrolizumab, followed by doxorubicin + cyclophosphamide + pembrolizumab; ACT: pembrolizumab vs. docetaxel + carboplatin + pembrolizumab; ACT: pembrolizumab	RCT; parallel assignment; open label	15 September 2023	N/A
NCT05978648	Trilaciclib in Patients with Early-Stage HR-Negative Breast Cancer Receiving Adjuvant Chemotherapy	2	trilaciclib + epirubicin + cyclophosphamide + paclitaxel	NR; single group; open label	20 September 2023	N/A
NCT06225284	Neoadjuvant Chemotherapy with or Without GnRH Agonist for Premenopausal Triple-negative Early Breast Cancer Patients	2	GnRH: goserelin or leuprolide or triptorelin + anthracycline + cyclophosphamide, followed by taxane and optional pembrolizumab vs. anthracycline + cyclophosphamide, followed by taxane and optional pembrolizumab	RCT; parallel assignment; open label	22 August 2024	N/A
NCT06795503	Non-Inferiority Study on MRNA-lncRNA Model in Low-Risk Triple-Negative Breast Cancer Patients	3	docetaxel + cyclophosphamide vs. epirubicin + cyclophosphamide, followed by paclitaxel	RCT; parallel assignment; open label	27 January 2025	N/A

RCT: randomized controlled trial; ACT: adjuvant chemotherapy.

### 6.4. Adaptive Therapy and Dosing Strategies

Inspired by evolutionary principles, researchers have tested non-traditional dosing schedules to manage resistance. Instead of the maximum tolerated dose (MTD) given on a fixed schedule, adaptive therapy adjusts the dosing based on tumour response to maintain a stable tumour burden and keep sensitive cells alive to suppress resistant cells [59]. For example, an adaptive regimen might provide docetaxel until a certain shrinkage is achieved, then stop or lower doses to allow sensitive cells to recover and outnumber resistant cells, and then resume treatment. This method has shown promise in prostate cancer models with hormone therapy, and trials for other cancers are in progress. Such strategies for breast cancer would be highly experimental; however, mathematically, they could delay the emergence of dominant–resistant populations. Additionally, dose-dense schedules (providing the same total dose in a shorter interval) have been tested clinically to outpace resistance. In early breast cancer, dose-dense taxane schedules improved survival in some studies, potentially by reducing the regrowth of resistant clones between cycles.

### 6.5. Biomarker-Guided Therapy

There has been a push towards personalised chemotherapy. Rather than assuming that all patients receive the same regimen, new trials should incorporate biomarkers of likely resistance to decide on therapy. For example, patients whose tumours show high TUBB3 or high MDR1 might be initially triaged away from docetaxel to an alternative (maybe to an epothilone or a platinum, etc.), or an added agent might be administered to counteract the specific resistance (e.g., a P-gp inhibitor in a clinical trial context or a PI3K inhibitor for a PIK3CA-mutant, Akt-activated tumour). Tests such as Oncotype DX currently guide the use of chemotherapy in general; future refinements may guide the selection of the chemotherapy type or treatment pairings. Researchers are validating assays for measuring expression of resistance-related genes (like an “MDR1 score” or a “stemness signature”) to predict chemo response. Interesting biomarkers include circulating tumour cells (CTCs) and circulating tumour DNA. A high burden of CTCs after a few cycles of chemotherapy can indicate early resistance, prompting the switch to alternate therapies before clinical progression.

### 6.6. Targeting the Tumour Microenvironment

Because the environment contributes to resistance (e.g., fibrosis-limiting drug delivery and hypoxia-inducing resistance genes), some approaches aim to modify the microenvironment. For instance, trials on TGF-β inhibitors (to prevent fibrosis/EMT) and CXCR4 inhibitors (to disrupt protective niches) are ongoing. Normalising tumour vasculature with agents such as bevacizumab (anti-VEGF) might improve docetaxel drug delivery, although in practice, the benefit of bevacizumab in breast cancer has been debated. Nevertheless, understanding that resistance is not purely tumour cell-autonomous will lead to holistic strategies.

### 6.7. Emerging Drug Targets from Omics

High-throughput screening identifies novel mediators of resistance. A recent CRISPR knockout screen in breast cancer cells revealed that the loss of certain epigenetic regulators makes cells more sensitive to docetaxel, indicating that these regulators are potential drug targets. Similarly, metabolomic studies have shown that resistant cells exhibit distinct metabolic pathways (e.g., greater dependence on oxidative phosphorylation). Inhibiting mitochondrial respiration with drugs such as metformin or specific OXPHOS inhibitors can preferentially kill chemoresistant cells that rely on that pathway [121]. Fascin itself was identified through unbiased approaches as a top hit connecting metastasis and resistance [62], validating the approach.

In the realm of fascin and actin dynamics, small molecules that disrupt cytoskeletal adaptations in resistant cells have been actively investigated. Compounds that target actin regulators or mitotic spindle assembly checkpoints (such that resistant cells that slip through mitosis can be caught by different mechanisms) are currently being studied.

### 6.8. Clinical Rechallenge and Sequencing

For patients, a pragmatic question is whether a cancer becomes resistant to docetaxel or whether it is permanently resistant. Interestingly, some data suggest that after a “drug holiday”, tumours might regain sensitivity (if the resistant population wanes because of fitness costs). A small clinical study on docetaxel rechallenge in metastatic breast cancer showed that a subset of patients responded to the reintroduction of docetaxel after having progressed previously [172]. The responses were not as high as those of the initial therapy; however, this raises the point that resistance is dynamic. Clinicians are also exploring alternative therapies to maintain tumour off-balance (e.g., alternating taxanes with another agent every few cycles). Such strategies are still experimental but underscore the evolving mindset in the treatment of drug-resistant cancers.

In conclusion, the period 2020–2025 saw significant progress in decoding docetaxel resistance. We appreciate this as a multidimensional problem involving genetics, epigenetics, non-coding RNAs, cellular phenotypes (such as EMT/CSC), and microenvironmental factors. Combating resistance requires combination approaches that target the structure of cancer cells (microtubules), their survival signals, and their interactions with the environment. Trials are becoming increasingly biomarker-driven to test these combinations in patient subgroups. It is hoped that by integrating these new insights, future therapies will either prevent resistance from developing or convert resistant diseases into chronic, manageable conditions. Ongoing research, including clinical trials of novel agents and treatment strategies, will determine how closely this goal is achieved (Table 1, Table 2, Table 3, Table 4 and Table 5). As our understanding deepens, the outlook for overcoming docetaxel resistance in breast cancer appears cautiously optimistic, moving us closer to more durable responses and improved survival in patients facing this challenge.

### 6.9. Nanoparticle-Based Strategies to Overcome Taxane Resistance: Advances, Benefits, and Current Challenges

Nanoparticle-based drug delivery systems offer promising solutions to enhance taxane efficacy, reverse resistance, and reduce systemic toxicity. Compared to conventional formulations, nanoparticles improve solubility and eliminate the need for toxic solvents, such as Cremophor EL or polysorbate 80, which are associated with hypersensitivity and require corticosteroid premedication. Systems such as nab-paclitaxel [173] and RNA-based nanoassemblies [174] increase the aqueous solubility and improve pharmacokinetics. Table 7 provides a comparative overview of docetaxel delivery systems in breast cancer.

In order to overcome failures encountered in clinical trials, nanoformulations offer a promising approach, as they can bypass drug efflux pumps, improve cellular uptake, and co-deliver agents that modulate drug resistance pathways. For instance, folate-targeted paclitaxel–fisetin nanoparticles downregulated ABCG2 and triggered apoptosis in resistant ovarian cells [178], whereas NanoOrl, an orlistat-loaded formulation, restored taxane sensitivity via fatty acid synthase inhibition, independent of P-gp in prostate cancer [179]. Docetaxel–resveratrol micelles demonstrated synergistic cytotoxicity against MCF-7 breast cancer cells, enhanced drug uptake, and prolonged circulation time in vivo, compared with the results for free drugs [180,181], and docetaxel–curcumin nanoparticles increased uptake, ROS generation, and apoptosis in MCF-7/Adr cells [182].

Controlled-release formulations further prolong intratumoural drug exposure. Hybrid PLGA-lipid nanoparticles reduced paclitaxel IC_50_ > 300-fold in anoikis-resistant lung cancer cells [183], and rod-shaped PLGA nanoparticles loaded with docetaxel improved survival and tumour inhibition in taxane-resistant TNBC mouse models [184].

Despite these advances, most nanoformulations remain in the preclinical stage. The challenges include poor scalability, limited in vivo validation, and regulatory uncertainty. As Patel et al. noted, many in vitro models fail to recapitulate tumour complexity, limiting clinical translation [185]. Even approved systems such as nab-paclitaxel offer only modest clinical benefits and raise cost-effectiveness concerns [173].

**Figure 5:**
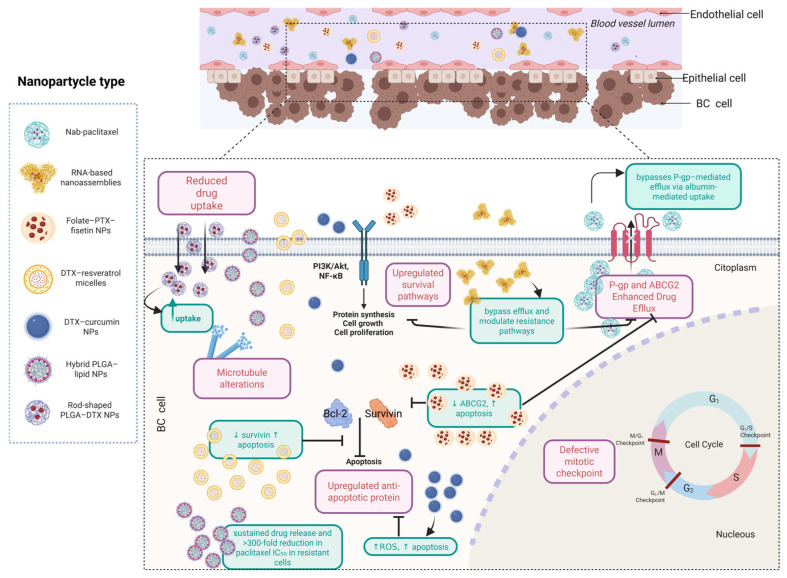
Nanoparticle-based strategies to overcome docetaxel resistance in breast cancer cells. This schematic illustrates how modern, nanoparticle-based delivery systems enhance docetaxel efficacy by circumventing key resistance mechanisms in breast cancer. Nanocarriers such as albumin-bound nanoparticles (e.g., nab-paclitaxel), polymeric micelles, and lipid–polymer hybrids improve drug uptake through passive or active targeting and avoid P-gp-mediated efflux. Some formulations (e.g., RNA-based nanoassemblies or folate-targeted combinations) modulate resistance pathways, suppress anti-apoptotic proteins (e.g., Bcl-2, survivin), or restore apoptotic signalling. Others sustain intracellular drug retention, reduce microtubule alterations, or inhibit survival pathways (e.g., PI3K/Akt, NF-κB). Collectively, these strategies contribute to overcoming multidrug resistance, enhancing tumour cytotoxicity, and potentially delaying treatment failure.

Nevertheless, innovations such as stimuli-responsive nanoparticles, surface-functionalized systems, and biodegradable carriers have expanded the therapeutic potential of these platforms [186]. Figure 5 illustrates the key strategies by which targeted nanoparticles overcome resistance, and Table 8 and Table 9 compare delivery systems currently under investigation in preclinical and clinical trials, respectively.

## 7. Conclusions and Perspectives

Docetaxel has indisputably improved the outcomes of breast cancer therapy; however, the emergence of resistance remains a formidable hurdle that limits its full potential. Resistance to docetaxel is a multifactorial phenomenon; tumour cells evade the effects of the drug through alterations in the drug target (tubulin/microtubules), active efflux of the drug, suppression of apoptotic pathways, adoption of an EMT and stem-like state, and various genetic/epigenetic adaptations. The actin-bundling protein fascin exemplifies how a single molecular change can simultaneously drive tumour aggressiveness and therapy resistance, making it an attractive target for next-generation treatments. An evolutionary perspective sheds light on how resistance develops, why a one-dimensional treatment approach is often inadequate, and the adaptability of cancer cells to adjustable therapeutic strategies.

The battle against docetaxel resistance is being waged on multiple fronts. Advances in genomic and transcriptomic profiling have pinpointed new mediators of resistance, particularly in the non-coding RNA realm. Therapeutic innovations, from novel taxane analogues to combinations of chemotherapy with targeted inhibitors or immunotherapy, are under active investigation in outsmart-resistant cancer cells. Early results suggest that tackling resistance mechanisms (such as using a PI3K inhibitor to counter Akt-driven survival, or a drug like bufalin to inhibit P-gp) can restore docetaxel sensitivity in preclinical trials [83]. In parallel, clinical strategies, such as personalised treatment regimens based on tumour biomarkers and adaptive dosing schedules, are being explored to delay or prevent evolution of resistance in patients.

Therefore, further research is required. Key areas for future studies include the discovery of reliable biomarkers that predict resistance (so therapy can be tailored accordingly), the development of safe inhibitors of proteins, such as fascin or survivin, that cancer cells use to resist apoptosis, and the integration of mathematical oncology models to optimise treatment sequencing. Moreover, understanding how the tumour microenvironment and host immune system contribute to chemoresistance may open new avenues (for example, using agents to modulate the stroma or combining chemotherapy with immunotherapies to target residual resistant cells).

In our view, targeting fascin holds exceptional therapeutic promise, as it functions both as a marker of tumour aggressiveness and as a mechanistic driver of resistance. Furthermore, incorporating dynamic biomarker monitoring (e.g., via serial liquid biopsies or ctDNA) may enable real-time treatment adaptation in clinical settings. We also advocate greater integration of evolutionary principles, such as adaptive dosing or temporally modulated combinations, into trial design, which may delay or prevent the emergence of resistant subclones.

In writing this narrative review, we underscored not only established concepts but also the most recent (2020–2025) findings in this fast-evolving field. The goal for clinicians and researchers is to convert our growing mechanistic knowledge into effective therapies that prevent or overcome docetaxel resistance, thereby prolonging remission and saving lives. With multidisciplinary efforts spanning molecular biology, pharmacology, and clinical trials, there is legitimate hope that what is now common clinical frustration (taxane resistance) will be a more manageable or even reversible condition in the future. The story of docetaxel resistance in breast cancer, once a seemingly intractable problem, is steadily being rewritten with chapters on innovation and improved understanding, moving us closer to the day when we can outsmart cancer resilience.

## Figures and Tables

**Table 1:** Predictive biomarkers of docetaxel resistance in breast cancer. This table summarises molecular and clinicopathological biomarkers associated with docetaxel resistance in breast cancer, as reported in peer-reviewed, recent PubMed-indexed studies. It includes both preclinical and clinical evidence.

Biomarker (Type)	Mechanism/Rationale	Evidence Type	Key Findings (Study Details)	References
ABCB1 (P-glycoprotein efflux pump)	Drug efflux transporter; overexpression reduces intracellular docetaxel response.	Preclinical	Overexpressed in resistant cells; inhibition restores sensitivity and drug accumulation; validated in mouse models.	[28,29,30]
EPB41L4A-AS2 (lncRNA)	Loss of tumour-suppressor lncRNA increases ABCB1 expression.	Preclinical	Absent in resistant cells; low expression associated with poor response to docetaxel.	[31]
LINC00667 (exosomal lncRNA)	Sponges miR-200b-3p to upregulate BCL2, reducing apoptosis.	Preclinical	Found in resistant TNBC exosomes; downregulation sensitises cells to docetaxel.	[32]
circABCB1 (circular RNA)	CircABCB1 contributed to the docetaxel resistance of breast cancer, possibly via miR-153-3p sponging.	Preclinical	The overexpression of circABCB1 contributed to cell viability, docetaxel-resistance, and migration/invasion.	[33]
circUBR5 (circular RNA)	circUBR5 sponges miR-340-5p, releasing suppression of CMTM6 and promoting c-MYC-driven ribosome biogenesis, contributing to docetaxel resistance.	Preclinical	Knocking down circUBR5 increased miR-340-5p activity, decreased CMTM6 levels, suppressed c-MYC activity, and sensitized cells to docetaxel—inducing apoptosis and reducing colony formation.	[34]
HORMAD1 (protein)	Promotes DNA damage tolerance via enhanced homologous recombination repair and protective autophagy, reducing docetaxel-induced apoptosis.	Preclinical	HORMAD1 overexpression protects TNBC cells from docetaxel-induced DNA damage and apoptosis; its knockdown restores chemosensitivity via impaired DNA repair and enhanced apoptosis.	[35]
TUBB3 (βIII-tubulin)	Alters microtubule dynamics, reducing docetaxel binding.	Preclinical + Clinical	High expression correlates with poor docetaxel response; inversely related to sensitivity.	[22,36]
METTL3 (RNA methyltransferase), LINC00662 (lncRNA)	Promotes docetaxel resistance by m6A-dependent stabilisation of LINC00662, forming a feedback loop with miR-186-5p that sustains METTL3 expression.	Preclinical	High METTL3 and LINC00662 levels were observed in docetaxel-resistant TNBC cells and patient samples; disrupting the METTL3/LINC00662/miR-186-5p axis restored chemosensitivity and increased apoptosis.	[37,38]
BAD (pro-apoptotic protein)	Facilitates necroptosis during prolonged mitotic arrest induced by docetaxel, preventing mitotic slippage and survival of resistant cells.	Preclinical	BAD expression promotes mitotic arrest and necroptotic death upon docetaxel treatment; its loss enables mitotic slippage and survival of chemoresistant polyploid cells. Tumours with high BAD show better response to taxanes.	[39]
ER/PR-positive status	Hormone-driven, low-proliferation tumours less responsive to taxanes.	Clinical	Associated with lower pCR rates compared to ER-negative tumours.	[40] and others
HER2-positive status	High proliferation and HER2-targetability improve taxane response.	Clinical	HER2+ tumours respond well to docetaxel-based regimens with HER2 inhibition.	[40] and others
Triple-negative subtype	High initial sensitivity; prone to relapse if pCR not achieved.	Clinical	Higher pCR rates but vulnerable to resistance upon incomplete response.	[40] and others

**Table 7:** Comparison of delivery systems in breast cancer (2020–present).

Delivery System	Formulation Type	Mechanism	Pros	Cons	Reference
Liposomes	Lipid bilayer vesicles (~50–200 nm) encapsulating drugs (e.g., PEGylated liposomal doxorubicin).	Passive tumour targeting via EPR; PEGylation (“stealth”) extends circulation; can be functionalized with ligands for active targeting.	Biodegradable, biocompatible; carry both hydrophilic and hydrophobic drugs; protect drug, improve pharmacokinetics (prolonged half-life, stability) and reduce systemic toxicity.	Rapid clearance by mononuclear phagocyte system without PEGylation; potential premature drug leakage and short circulation half-life; high manufacturing cost.	[175]
Polymeric NPs	Biodegradable polymer nanoparticles (e.g., PLA/PLGA nanospheres or nanocapsules loaded with paclitaxel—PICN^®^ is a polymeric PTX NP approved in India for metastatic breast cancer).	Polymer matrix entraps drug and releases it via controlled degradation; passive EPR targeting (with possible ligand-mediated active targeting).	Highly versatile (wide choice of polymers); stable during storage and scalable manufacturing; tunable surface properties and drug release; high drug payload capacity; improve bioavailability and circulation time.	Possible stability issues (tendency to aggregate); require precise manufacturing conditions; some formulations need PEGylation for prolonged circulation; potential toxicity of residual monomers or solvents.	[176]
Polymeric Micelles	Self-assembled amphiphilic copolymer micelles (10–100 nm) solubilizing hydrophobic drugs in a core (e.g., PEG-PLA micelle paclitaxel, Genexol-PM^®^).	Spontaneous micelle formation above a critical micelle concentration; drugs carried in core are released upon micelle dissociation or stimulus in tumour microenvironment.	Easy to prepare; improve water solubility of hydrophobic drugs; prolong circulation and enhance tumour accumulation via EPR; increase drug efficacy and reduce toxicity (no need for harsh solubilizers like Cremophor)	Limited stability in bloodstream—dilution below critical micelle concentration causes disassembly and rapid drug clearance (short half-life in circulation).	[177]
Albumin-Bound NPs	Albumin-based nanoparticles or albumin–drug complexes (e.g., nab-Paclitaxel, Abraxane^®^ ~130 nm, an albumin-bound paclitaxel approved for metastatic breast cancer).	Exploit albumin’s natural pathways: passive tumour accumulation via EPR and active transcytosis (gp60 receptor) and binding to SPARC in tumour stroma, enhancing drug delivery to tumour sites.	Biocompatible, non-immunogenic carrier; avoids toxic solvents (Abraxane is Cremophor-free); long circulation and tumour uptake via albumin receptors; improves drug solubility and bioavailability	Require cross-linking for nanoparticle stability (e.g., glutaraldehyde crosslinker, which can leave toxic residues); net negative charge of albumin can limit drug loading unless chemically modified.	[175]

**Table 8:** Nanoparticle-based strategies to overcome docetaxel resistance in breast cancer preclinical studies (2020–2025). This table summarises peer-reviewed, PubMed-indexed preclinical studies published between 2020 and 2025 that investigated nanoparticle-based strategies to overcome docetaxel resistance in breast cancer. It includes information on nanoparticle type, therapeutic cargo, targeting strategies, resistance mechanisms addressed, key findings, development stage, and references.

Nanoparticle Type	Cargo (Co-Delivered Agents)	Targeting Strategy	Resistance Mechanism Addressed	Key Findings	Stage	Reference
pH-sensitive PLGA nanoparticle	Docetaxel + Disulfiram (DSF)	pH-triggered release, TPGS-mediated P-gp inhibition	P-gp efflux, CSC survival, tumour stroma barrier	Restored sensitivity in resistant cells, enhanced tumour accumulation, inhibited metastasis, superior efficacy in vivo	Preclinical	[187]
Liposome (CUR-DTX-L)	Docetaxel + Curcumin	Passive targeting (EPR)	MDR via efflux transporters, survival signalling	Synergistic cytotoxicity, prolonged half-life, tumour growth inhibition in xenograft model	Preclinical	[188]
RGD-decorated PLGA nanoparticle	Docetaxel (+ MRI/fluorescent tracers)	αvβ3 integrin targeting	Limited tumour uptake, systemic toxicity	Higher tumour localization, reduced cardiotoxicity, improved efficacy in TNBC and HER2+ models	Preclinical	[189]
PLGA–TPGS polymeric nanoparticle	Docetaxel	Passive targeting, TPGS-mediated P-gp inhibition	General MDR, poor intracellular accumulation	Increased potency, reduced IC_50_, sustained release, improved anti-proliferative effect	Preclinical	[190]
Solid lipid nanoparticle (SLN)	Docetaxel	Passive targeting, controlled release	EMT, IL-6/BCL-2 survival signalling	High cytotoxicity, G2/M arrest, prevented metastasis, suppressed IL-6 and BCL-2	Preclinical	[191]
Folate-targeted pH/ROS-dual responsive nanoparticle	Docetaxel + Cinnamaldehyde	Folate receptor targeting, stimuli-responsive release	TNBC metastasis, immune evasion	Immunogenic cell death, blocked invasion, halted metastasis, enhanced anti-PD-1 response	Preclinical	[192]
Exosome-coated polyamine nanocomplex	Docetaxel + miR-34a	Biomimetic targeting (exosomal membrane)	miR-34a loss, anti-apoptotic signalling	High cytotoxicity, BCL-2 downregulation, potent apoptosis induction	Preclinical	[193]
Lipid-coated mesoporous silica nanoparticle (LP-MSN)	Docetaxel + Tamoxifen	Sequential release (Tamoxifen then DTX)	CYP3A4-mediated metabolic resistance	Enhanced cytotoxicity via CYP3A4 inhibition, selective toxicity to TNBC cells	Preclinical	[194]

**Table 9:** Nanoparticle-based docetaxel strategies in breast cancer clinical trials. This table summarizes Phase I–III clinical trials registered between 2020 and 2025 on nanoparticle-based docetaxel strategies aimed at overcoming resistance in breast cancer. Data are sourced from ClinicalTrials.gov and EudraCT. Only human studies are included.

Trial Identifier	Nanoparticle Formulation	Combination Therapy	Targeting Strategy/Delivery Type	Phase	Status	Objective Summary
NCT03671044	Nanosomal Docetaxel Lipid Suspension (NDLS)	None (monotherapy)	Lipid-based, polysorbate-free formulation to improve solubility and tumour delivery	Phase 3	Recruiting	Compare efficacy and safety of NDLS vs. conventional docetaxel in TNBC patients resistant to prior chemotherapy.
NCT04931823	CPO-100 (Albumin-bound Docetaxel)	None	Albumin nanoparticle, solvent-free to enhance safety and tumour targeting	Phase 1	Active	Evaluate MTD, safety, PK, and preliminary efficacy in advanced solid tumours refractory to standard treatment, including breast cancer.
NCT05114915	Albumin-bound Docetaxel (HB1801)	None	Albumin-stabilized nanoparticle, solvent-free for safer delivery	Phase 1	Recruiting	Assess safety, tolerability, PK, and preliminary efficacy in advanced solid tumours unresponsive to standard therapies (includes breast cancer).
NCT05254665	Polymeric Micellar Docetaxel	None	Polymeric micelle nanoparticle for improved tumour-specific delivery	Phase 2	Not yet recruiting	Confirm dose, assess safety and anti-tumour efficacy in taxane-resistant advanced solid tumours, including breast cancer.

## Abbreviations

The following abbreviations are used in this manuscript:

AKT	Protein Kinase B
ALDH	Aldehyde Dehydrogenase
ATP	Adenosine Triphosphate
BAD	BCL2 Associated Agonist of Cell Death
BAK	BCL2 Antagonist/Killer
BAX	BCL2 Associated X Protein
BCL	B-Cell Lymphoma
BCRP	Breast Cancer Resistance Protein
CR	Complete Response
CRISPR	Clustered Regularly Interspaced Short Palindromic Repeats
CSC	Cancer Stem Cell
DNA	Deoxyribonucleic Acid
DTX	Docetaxel
EGF	Epidermal Growth Factor
ER	Estrogen Receptor
ERK	Extracellular Signal-Regulated Kinase
HER2	Human Epidermal Growth Factor Receptor 2
HIF	Hypoxia-Inducible Factor
IGF	Insulin-like Growth Factor
IKK	IκB Kinase
JAK	Janus Kinase
MAP	Microtubule-Associated Protein
MAPK	Mitogen-Activated Protein Kinase
MEK	Mitogen-Activated Protein Kinase
MMP	Matrix Metalloproteinase
MYC	Myelocytomatosis Viral Oncogene
NF	Nuclear Factor
OXPHOS	Oxidative Phosphorylation
PARP	Poly (ADP-Ribose) Polymerase
PD	Progressive Disease
PF	Progression-Free
PR	Progesterone Receptor
PTEN	Phosphatase and Tensin Homolog
RAF	Rapidly Accelerated Fibrosarcoma
RAS	Rat Sarcoma Viral Oncogene Homolog
RNA	Ribonucleic Acid
ROR	Regulator of Reprogramming
SMAC	Second Mitochondria-Derived Activator of Caspases
TGF	Transforming Growth Factor
VEGF	Vascular Endothelial Growth Factor
XIAP	X-linked Inhibitor of Apoptosis Protein
